# Unfermented High‐Fiber Rye Crispbread Increases Plasma HDL and Reduces Hepatic Lipids Compared to Refined Wheat in Rats Fed a High‐Fat Diet

**DOI:** 10.1002/mnfr.70352

**Published:** 2025-12-13

**Authors:** Fidèle Almasri, Eleonora Aimaretti, Nadine Sus, Erik Schéle, Suzanne L. Dickson, Rikard Landberg, Massimo Collino, Jan Frank

**Affiliations:** ^1^ Department of Food Biofunctionality Institute of Nutritional Sciences, University of Hohenheim Stuttgart Germany; ^2^ Department of Neurosciences “Rita Levi Montalcini” University of Turin, Corso Raffaello Torino Piemonte Italy; ^3^ Department of Physiology Institute of Neuroscience and Physiology The Sahlgrenska Academy at the University of Gothenburg Gothenburg Sweden; ^4^ Department of Life Sciences Division of Food and Nutrition Science Chalmers University of Technology Gothenburg Sweden

**Keywords:** body weight, lipid metabolism, rye, wheat

## Abstract

Fiber‐rich rye foods reduced body weight in overweight or obese individuals compared to refined wheat, though the underlying mechanisms remain unclear. This study compared the effects of whole grain fermented and unfermented rye with refined wheat crispbread on body weight and lipid metabolism in rats. Exploratory outcomes included adiposity, appetite biomarkers, glucose homeostasis, colon inflammation, integrity, and permeability. Sprague Dawley rats (*n* = 54) were acclimatized (2 weeks) and randomized to control (*n* = 9) or high‐fat diets (*n* = 45) for 16 weeks. Animals in the high‐fat group were randomized to continue the high‐fat (*n* = 9) or receive a standard diet alone (*n* = 9) or the standard diet with either refined wheat crispbread (*n* = 9), fermented rye crispbread (*n* = 9), or unfermented rye crispbread (*n* = 9) for 8 weeks. A high‐fat diet did not affect final body weight, glucose homeostasis, and colon inflammation, but increased energy intake, adiposity, and leptin compared to control, and hepatic triacylglycerols compared to all other groups. Unfermented rye crispbread increased plasma HDL‐cholesterol and reduced hepatic triacylglycerols and cholesterol compared to refined wheat, despite the absence of effects on obesity and glycemic control. No differences were observed between fermented and unfermented rye. Unfermented whole‐grain rye crispbread may potentially exhibit favorable lipid‐modulating effects.

## Graphical abstract

This study compared the effects of whole grain fermented and unfermented rye crispbread with refined wheat on body weight and lipid metabolism in rats. Unfermented rye crispbread increased plasma HDL‐cholesterol and reduced hepatic triacylglycerols and cholesterol compared to refined wheat, indicating favorable lipid‐modulating effects.

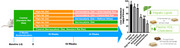

## 1 Introduction

Obesity is a global epidemic and a main driver of metabolic disorders, such as cardiovascular diseases and type 2 diabetes mellitus (Seventy‐fifth World Health Assembly [1]), which is why research into the prevention and treatment of obesity is of increasing importance. Epidemiological studies have found high intake of whole grain cereals to be associated with improved long‐term weight control [2], which has been attributed to their high content of dietary fiber. In humans, the consumption of dietary fiber in general [3] and of fiber‐rich rye foods in particular leads to increased satiety and dampened blood glucose and insulin responses compared to refined wheat foods [4]. These effects are attributed to the digestive behavior of dietary fibers, particularly arabinoxylan in whole‐grain rye, which has been shown in vitro to absorb water, swell, and form viscous solutions that slow gastric emptying, delay glucose absorption, and promote satiety [5]. However, in vitro studies also suggest that fermentation of rye bread may degrade dietary fibers, reduce their viscosity, and increase glucose absorption [6], but in vivo evidence remains scarce. The microstructure of starchy foods, which can be modified by processing, is another important determinant of the fiber physiological effects; for example, consumption of more coarsely ground flours leads to lower postprandial blood glucose and insulin responses compared to finely ground flours [7]. These observations provide a rationale to investigate whether processing, such as fermentation, may modulate the physiological effects of rye food consumption.

A 12‐week hypocaloric diet with high‐fiber rye foods (corresponding to about 30% of the daily energy intake) reduced body weight and obesity, modulated gut microbiota composition, and increased plasma butyrate, a fermentation by‐product, when compared to refined wheat in an obese population [8, 9], suggesting that a daily intake of high‐fiber rye may lead to larger weight loss compared with similar isocaloric intake of refined wheat, possibly via improved satiety and metabolic control.

To investigate underlying mechanisms that are difficult to assess in humans, we conducted a controlled study in a model of Sprague‐Dawley rats, and examined the effects of whole‐grain fermented and unfermented rye crispbread compared to refined wheat crispbread on body weight as the primary outcome, and further obesity‐related metabolic biomarkers such as appetite and glycemia as exploratory outcomes.

## 2 Materials and Methods

### 2.1 Animals and Study Design

Animal procedures were performed in accordance with the Federation of European Laboratory Animal Science Association guidelines for care and use of laboratory animals, and ethical approval was received from the Regional Council (Stuttgart, Baden‐Württemberg; RPS35‐9185‐99/417). Sprague‐Dawley rats were selected for this study as an established model used in metabolic studies, favored for their ease of handling, large blood volume, and similarities to humans in responsiveness to feeding cues [10, 11].

**FIGURE 1:**
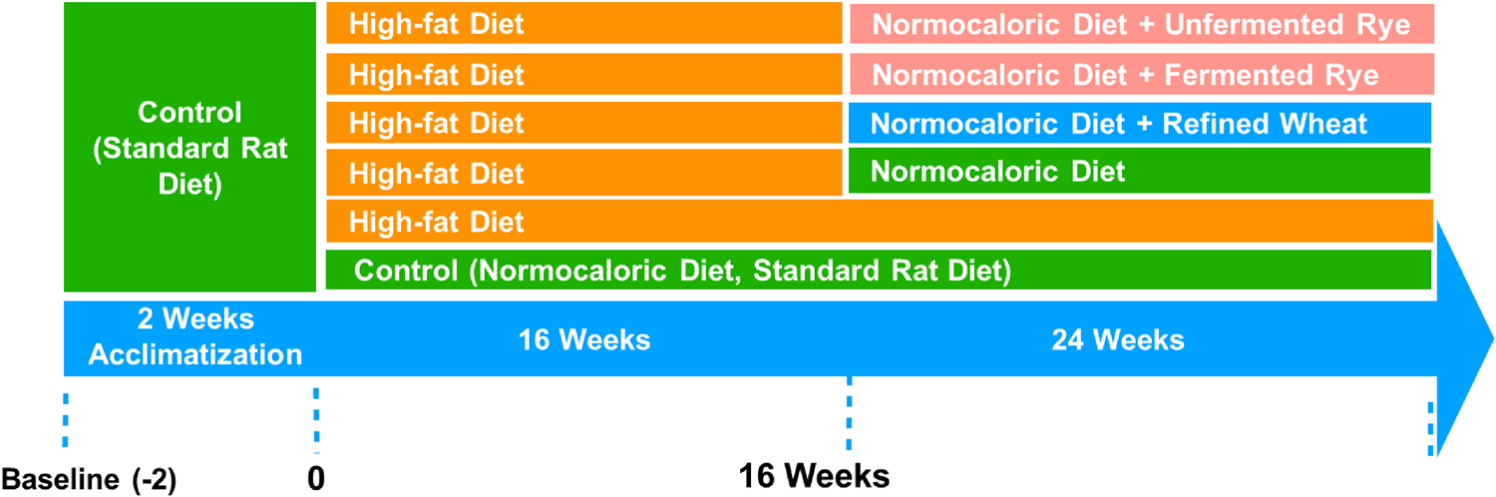
Study design: Rats (*n* = 54) were acclimatized on a standard rat diet for 2 weeks and then randomly assigned to a control (*n* = 9) or high‐fat diet (*n* = 45) for 16 weeks. Then the animals in the high‐fat group were randomized to continue the high‐fat diet (*n* = 9) or to receive a standard diet alone (*n* = 9), or the standard diet with either refined wheat crispbread (*n* = 9), fermented rye crispbread (*n* = 9) or unfermented rye crispbread (*n* = 9) for 8 weeks.

Fifty‐four male Sprague‐Dawley rats (250–270 g) were purchased from Janvier Labs (Le Genest St. Isle, France) at 6 weeks of age and familiarized with the animal facility and fed a standard diet for 15 days. Then the animals were randomly assigned to a control group (9 animals) fed a normocaloric standard diet for the entire study period (24 weeks) or a high‐fat group (45 animals) receiving a diet with 60% of total energy derived from lard for 16 weeks to induce weight gain (Figure 1). Following the 16‐week high‐energy diet phase, rats in the high‐fat group were randomized (with stratification of body weight to ensure an equal distribution across the groups) to one of five different groups. One group continued the high‐fat diet (high‐fat control; *n* = 9), and the remaining four groups were switched from a high‐fat diet to a standard rat diet to simulate a normocaloric dietary regimen for 8 more weeks. One group was fed the standard control diet alone (high‐fat→control; *n* = 9), one the standard diet plus refined wheat crispbread (*n* = 9), one the standard diet plus unfermented rye crispbread (*n* = 9), and one the standard diet plus fermented rye crispbread (*n* = 9; Figure 1). The pelleted feed and the crispbreads were placed on the top of the cages, and access to feed and water was unrestricted. Feed and crispbread intake were recorded daily, and body weight was measured weekly. The feed was from Altromin Spezialfutter GmbH & Co. KG (Lage, Germany), and crispbreads were from a previous human randomized controlled trial [8] and accounted for one‐third of total daily feed intake (Table 1), ensuring there was no nutritional restriction. The ingredients of the refined wheat crispbread were: sifted wheat flour, yeast, and salt; of the whole‐grain rye crispbread: whole‐grain rye flour, yeast, and salt; and of the whole‐grain unfermented rye crispbread: whole‐grain rye flour and salt. Rats were housed three per cage and kept in a controlled environment at 20 ± 2°C, 12 h light/12 h dark cycle, and 55 ± 10% relative humidity.

**TABLE 1:** Macronutrient compositions of the diets and crispbreads used in the experiment.

Percentage energy of macronutrient	Standard rat diet	High‐fat diet	Refined wheat crispbread	Fermented rye crispbread	Unfermented rye crispbread
Fat (%)	10	60	18	6	4
Starch (%)	63	22	67	70	76
Fiber (%)	3	2	2	12	10
Protein (%)	24	16	13	12	10
Total energy (kcal/100 g)	348.63	525.41	392.4	335.6	336.1

Abbreviation: Kcal, kilocalorie.

At the end of the experiment, rats were fasted for 6 h, anesthetized by carbon dioxide gas, and euthanized by decapitation. Blood was collected into heparinized tubes for plasma and plain tubes for serum (Monovette, Sarstedt, Nümbrecht, Germany) and placed on ice. Plasma and serum were separated from the blood cells within 2 h after collection. Whole blood was separately collected in a tube with a protease inhibitor for ghrelin analysis. Tissues were excised, placed in cryotubes, and snap‐frozen in liquid nitrogen. Samples were stored at −80°C until analysis. Freeze‐thaw cycles were avoided during sample handling.

### 2.2 Biomarkers of Appetite and Glucose Homeostasis

Fasting plasma leptin and acyl‐ghrelin were measured in duplicate using commercial enzyme‐linked immunosorbent assays (ELISA). Leptin was analyzed following the manufacturer's instructions (BIOZOL Diagnostica Vertrieb GmbH, Hamburg, Germany; kit no. BVD‐RD291001200R‐96), whereas acyl‐ghrelin measurement followed both the manufacturer's protocol (EZRGRA‐90K; Merck KGaA, Darmstadt, Germany), and a previously established method [12].

Fasting blood glucose was measured from a drop of fresh blood using a glucometer (TESTAmed; Diabetes care, Germany), and fasting plasma insulin was analyzed with ELISA (BVD‐RAI008R‐96; BIOZOL).

### 2.3 Plasma and Liver Lipids

Plasma triacylglycerols (cat. no. CL53‐200S), cholesterol (cat. no. CL21‐200S), and HDL‐PEG cholesterol (cat. no. CL22‐160) were measured using commercially available kits (FAR Diagnostics, Verona, Italy). Non‐HDL cholesterol was calculated as follows: non‐HDL cholesterol = total cholesterol‐HDL cholesterol [13].

To quantify hepatic triacylglycerols and cholesterol, liver lipids were extracted by preparing a 10% (w/v) tissue homogenate in 5% Triton X‐100 in distilled water using the gentleMACS Octo tissue dissociator (Miltenyi Biotec B.V. & Co. KG, Gladbach, Germany; protocol: 53 s, 2753 RPR). The homogenate was then heated at 90°C for 5 min and cooled on ice for 5 min, repeated twice. Next, samples were centrifuged at 20854 RCF for 10 min at room temperature using an Eppendorf Centrifuge 5810R (Eppendorf SE, Hamburg, Germany), and the supernatants were collected. Hepatic triacylglycerols and cholesterol were determined using the same kits as for plasma analyses.

Total hepatic lipids were quantified using the sulfo‐phospho‐vanillin assay, as previously described [14]. Liver tissue (30 mg) was homogenized in 500 µL of sodium phosphate buffer (PBS) using the gentleMACS tissue dissociator (protocol: 53 s, 2753 RPR). After brief centrifugation, samples were transferred to 2 mL tubes and mixed with a chloroform‐methanol solution (2:1, v/v). Following centrifugation at 4°C, 50 µL of the organic phase was evaporated at 90°C, cooled on ice, and treated with 100 µL of concentrated H_2_SO_4_ before reheating. Vanillin reagent was added to the samples, which were incubated at room temperature for 40 min. Finally, 200 µL aliquots were transferred to a 96‐well plate, and absorbance was read at 550 nm using a spectrophotometer (BioTek Synergy LX Multimode Reader, Agilent Technologies, Santa Clara, CA, USA).

### 2.4 Hepatic Inflammation

Myeloperoxidase (MPO) activity was assessed following a previously described method [15]. Briefly, 100 mg of liver tissue was homogenized and centrifuged at 13 000 RPM for 10 min at 4°C. MPO activity was determined by measuring the H_2_O_2_‐dependent oxidation of 3,3′,5,5′‐tetramethylbenzidine. Results were expressed as optical density at 650 nm per milligram of protein.

Relative mRNA expression in liver of C‐C motif chemokine ligand 2 (CCL2), C‐C motif chemokine ligand 5 (CCL5), and retinoic acid‐related orphan receptor C (RORc) was quantified by quantitative real‐time polymerase chain reaction (qPCR). In brief, mRNA was extracted, purity and concentration measured, and adjusted for a standard concentration to synthesize cDNA. The qPCR reaction was carried out using Bio‐Rad Real‐Time PCR systems, with optimized conditions for each target gene. Gene expression was normalized to 18s as a reference gene.

### 2.5 Biomarkers of Inflammation, Gut Integrity, and Permeability in the Colon

Relative mRNA expression in colon tissue of tumor necrosis factor alpha (TNF‐α), interleukin‐1β (IL‐1β), interleukin‐6 (IL‐6), zonula occludens (ZO‐1), and occludin (OCLN) was quantified by RT‐qPCR. Gene expression was normalized using ribosomal protein lateral stalk subunit P0 (RPLP0) and β2‐microglobulin (B2M) as reference genes. The methodology and reagents used in RT‐qPCR differed slightly between liver and colon tissues, as detailed in the Supporting Information (Tables S1–S3).

Plasma lipopolysaccharide (LPS) was analyzed in duplicates with sandwich ELISA (orb782514; Biorbyt, Cambridge, United Kingdom).

### 2.6 Statistical Analyses

Body weight was considered the primary outcome, and sample size was calculated with a power of 80% and a significance level of *p* < 0.05 to ensure detecting a difference in body weight of 15% with a standard deviation of 11%. This resulted in a minimum number of nine rats per group. Data analysis was conducted using GraphPad Prism 9.5 (GraphPad Software, San Diego, CA, USA). Normal distribution of the data was checked with the Shapiro–Wilk test, and the equal variance assumption was verified prior to applying analysis of variance (ANOVA). Changes in body weight and energy intake between the groups over the 26‐week period (2 weeks acclimatization and 24 weeks intervention) were analyzed using repeated measures ANOVA with Tukey post‐hoc test. Effects of diet at the endpoint were calculated using one‐way ANOVA with Tukey post‐hoc test. Statistical significance was set at *p* < 0.05, and results are presented as mean ± standard error of the mean (SEM). Data that were not normally distributed were log‐transformed and tested again for Gaussian distribution. If the Shapiro–Wilk test showed a *p* value > 0.05, the statistical analysis was performed by one‐way ANOVA, followed by Tukey's post‐hoc test. Non‐parametric statistical analysis was computed using the Kruskal–Wallis followed by Dunn's post hoc‐test on the set of data that remained non‐normally distributed, with a *p* value > 0.05 from Shapiro–Wilk test.

## 3 Results and Discussion

The aim of the present study was to investigate if the intake of dietary fiber and associated bioactive compounds from rye crispbread might facilitate weight loss in rats when switching from a high‐fat and high‐energy diet to a normocaloric diet. Because fermentation may change the properties of dietary fiber, we further studied potential differences in the effects of fermented whole grain rye and unfermented whole grain rye crispbreads, with refined wheat, which is low in dietary fiber.

### 3.1 Body Weight, Adiposity, and Energy Intake

**FIGURE 2:**
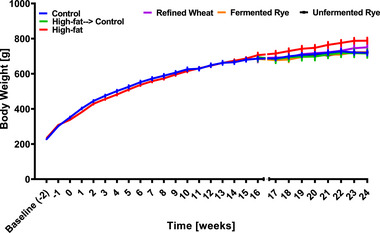
Mean body weight of rats. Rats (*n* = 54) were acclimatized on a standard rat diet for 2 weeks and then randomly assigned to a control (*n* = 9) or high‐fat diet (*n* = 45) for 16 weeks. Then the animals in the high‐fat group were randomized to continue the high‐fat diet (*n* = 9) or to receive a standard diet alone (*n* = 9), or the standard diet with either refined wheat crispbread (*n* = 9), fermented rye crispbread (*n* = 9) or unfermented rye crispbread (*n* = 9) for 8 weeks. Data are presented as mean ± SEM from nine biological replicates (individual rats) per group. Statistical analysis was performed using repeated measures ANOVA with Tukey post‐hoc test.

During the first phase of the study and following a 2‐week acclimatization period, the rats were fed either a normocaloric standard rat diet (control) or a hypercaloric high‐fat diet for 16 weeks (Figure 1), during which no significant differences in body weight were observed between groups (Figure 2). In the second phase (Week 17 onwards), the control group continued the standard diet, the high‐fat group continued the hypercaloric diet (high‐fat control), and the remaining animals were divided into four groups and fed either a standard diet or the standard diet plus either crispbread made from refined wheat, fermented rye, or unfermented rye (Figure 1). Animals on the high‐fat diet had numerically, but not significantly, higher body weight than control rats at the end of the experiment. Changing the hypercaloric to a normocaloric diet (high‐fat→control) resulted in a body weight similar to that of the animals fed a normocaloric diet throughout the entire study. The addition of crispbread to the standard diets did not further affect body weight (Figure 2).

**FIGURE 3:**
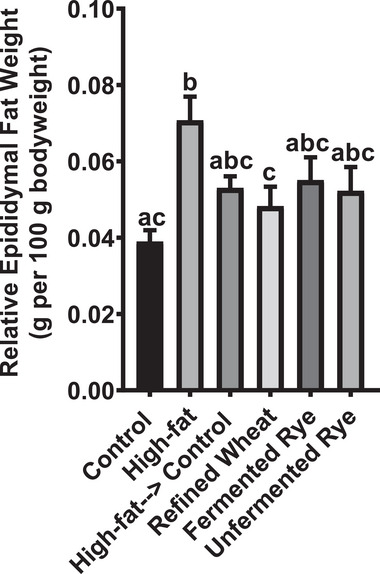
Mean relative epididymal fat weight (g per 100 g bodyweight) of rats at the end of the trial. Rats (*n* = 54) were acclimatized on a standard rat diet for 2 weeks and then randomly assigned to a control (*n* = 9) or high‐fat diet (*n* = 45) for 16 weeks. Then the animals in the high‐fat group were randomized to continue the high‐fat diet (*n* = 9) or to receive a standard diet alone (*n* = 9), or the standard diet with either refined wheat crispbread (*n* = 9), fermented rye crispbread (*n* = 9) or unfermented rye crispbread (*n* = 9) for 8 weeks. Data are presented as mean ± SEM from nine biological replicates (individual rats) per group. Statistical analysis was performed using repeated measures ANOVA with Tukey post‐hoc test. Bars not sharing a superscript letter are significantly different at *p* < 0.05.

Gonadal fat is a well‐established marker for total fat mass in rodent models [16]. Compared to control, animals fed the high‐fat diet had significantly increased relative epididymal fat (g/100 g body weight). Changing to a standard diet with or without crispbread‐feeding numerically reduced epididymal fat compared to high‐fat feeding, and significantly in the animals fed the refined wheat crispbread (Figure 3). This may be attributed to the reduction in energy intake or to potential human error during the collection of gonadal tissues, as slight numerical differences were observed among groups fed the standard rat diet with or without crispbread.

**FIGURE 4:**
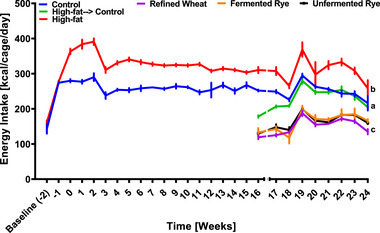
Mean energy intake per cage. Rats (*n* = 54) were acclimatized on a standard rat diet for 2 weeks and then randomly assigned to a control (*n* = 9) or high‐fat diet (*n* = 45) for 16 weeks. Then the animals in the high‐fat group were randomized to continue the high‐fat diet (*n* = 9) or to receive a standard diet alone (*n* = 9), or the standard diet with either refined wheat crispbread (*n* = 9), fermented rye crispbread (*n* = 9) or unfermented rye crispbread (*n* = 9) for 8 weeks. Data are presented as mean ± SEM from nine biological replicates (individual rats) per group. Statistical analysis was performed using repeated measures ANOVA with Tukey post‐hoc test. Mean values not sharing a superscript letter are significantly different at *p* < 0.05. Kcal, kilocalorie.

Feed intake did not differ between the control, high‐fat, and high‐fat→control groups, and consumption of crispbread did not differ between the groups fed with different crispbreads (Figure S1). Rats on the high‐fat diet consumed significantly more energy than rats on the control diet throughout the entire experiment (Figure 4). Switching from the high‐fat diet to a standard rat diet reduced energy intake significantly to the same level as the control group. Adding any type of crispbread to the standard rat diet resulted in a further significant reduction in energy intake compared to the standard diet alone, as well as the high‐fat group (Figure 4). No significant differences in energy intake were observed between the groups that were fed crispbread.

The observed resistance to diet‐induced obesity of our rats is a limitation and in agreement with previous findings that Sprague‐Dawley rats may exhibit resistance to diet‐induced obesity and its associated metabolic complications, such as adiposity, glucose intolerance, insulin resistance, and elevated circulating cholesterol [17, 18, 19, 20]. More rapid and pronounced metabolic responses to diet‐inducing obesity have been observed for Wistar rats, compared to Sprague‐Dawley rats, likely due to higher lipogenesis and fatty acid uptake [18], suggesting that they may be a more suitable model for diet‐induced obesity [18, 21, 22].

### 3.2 Biomarkers of Appetite and Glucose Homeostasis

Leptin is a peptide hormone primarily produced by white adipose tissue and acts on the brain to regulate energy homeostasis and suppress appetite [23]. Plasma concentrations of leptin were significantly increased at the end of the trial in the high‐fat group compared to the control group (Table 2), but switching from a high‐fat diet to a standard diet (high‐fat→control) did not significantly reduce leptin concentrations and neither did the additional consumption of crispbread. The significant induction of leptin by the high‐fat diet and the non‐significant, but numerical reduction of plasma leptin concentrations in all groups that were switched to the normocaloric standard diet in the second phase of the feeding trial, are in agreement with the changes observed in body fat, and consistent with the secretion of leptin by adipocytes and its function in signaling satiety. However, these trends should be interpreted with caution since the differences were not statistically significant.

**TABLE 2:** Plasma concentrations of satiety biomarkers, energy metabolism markers, lipids, and gut permeability markers in rats at the end of the trial.

	Control	High‐fat	High‐fat → Control	Refined wheat	Fermented rye	Unfermented rye
Leptin (ng/mL)	10.3 ± 1.5 ^a^	21.2 ± 2.5 ^b^	13.7 ± 2.0 ^ab^	17.5 ± 3.5 ^ab^	15.1 ± 2.4 ^ab^	12.3 ± 3.0 ^ab^
Active (acyl) ghrelin (pg/mL)	102.6 ± 17.2	76.00 ± 56.7	40.4 ± 4.3	72.9 ± 10.2	64.4 ± 26.5	290.9 ± 166.3
Fasting blood glucose (mg/dL)	93.3 ± 9.1	90.6 ± 4.9	105.1 ± 23.1	89.0 ± 10.6	90.7 ± 7.5	94.4 ± 8.6
Insulin (ng/dL)	0.7 ± 0.2	0.7 ± 0.2	0.8 ± 0.2	0.6 ± 0.2	0.9 ± 0.3	0.9 ± 0.3
Triacylglycerols (mg/dL)	143.5 ± 18.2	134.3 ± 11.6	216.0 ± 23.3	201.8 ± 17.3	189.5 ± 29.6	193.6 ± 13.6
Cholesterol (mg/dL)	109.5 ± 10.3	128.2 ± 12.7	123.6 ± 7.2	112.7 ± 8.3	129.1 ± 7.5	149.9 ± 13.3
HDL cholesterol (mg/dL)	36.9 ± 4.0 ^a^	49.0 ± 2.7 ^a^	46.1 ± 5.8 ^a^	53.4 ±4.4 ^ab^	53.4 ± 3.4 ^ab^	69.4 ± 5.0 ^b^
Non‐HDL cholesterol (mg/dL)	72.6 ± 11.1	67.6 ± 3.8	77.5 ± 6.3	58.0 ± 8.3	75.8 ± 6.3	80.5 ± 11.9
Lipopolysaccharide (pg/mL)	212 ± 20.6	265 ± 22	237 ± 16.3	242 ± 26.7	213 ± 18.3	292 ± 22.3

*Note*: Data are presented as mean ± SEM from nine biological replicates (individual rats) per group. Each sample was measured in duplicate (technical replicates). Statistical analysis was performed using one‐way ANOVA with Tukey post‐hoc test. Mean values not sharing a common superscript letter are significantly different at *p* < 0.05. Rats (*n* = 54) were acclimatized on a standard rat diet for 2 weeks and then randomly assigned to a control (*n* = 9) or high‐fat diet (*n* = 45) for 16 weeks. Then the animals in the high‐fat group were randomized to continue the high‐fat diet (*n* = 9) or to receive a standard diet alone (*n* = 9), or the standard diet with either refined wheat crispbread (*n* = 9), fermented rye crispbread (*n* = 9) or unfermented rye crispbread (*n* = 9) for 8 weeks.

Ghrelin is an orexigenic peptide hormone, secreted mainly by the stomach, and stimulates appetite by signaling hunger to the brain [24]. Plasma concentrations of active ghrelin did not significantly differ between groups (Table 2), and large variations between animals within each group were observed. Therefore, it is not possible to draw firm conclusions about the effects of a high‐fat diet on plasma ghrelin or the potential benefits of switching to a hypocaloric diet or adding crispbread.

Our findings align with the absence of significant differences in body weight and adiposity observed in our study (Figures 2 and 3). The high‐fat diet induced a moderate (but not significant) increase in body weight and a significant fat accumulation, which stimulated leptin secretion into the circulation. The results are in line with previous research showing that 17 weeks of high‐fat feeding induced a moderate increase in adiposity and plasma leptin concentration in Sprague Dawley rats, with more pronounced or earlier responses observed in Wistar rats receiving the same treatment [22].

On the other hand, the crispbread intervention in our study did not confer additional benefits in reducing body weight or adiposity and, consequently, did not appear to influence leptin release from white adipose tissue. In C57BL/6J mice, on the other hand, feeding a cake containing 11% lard fat supplemented with either whole grain wheat or rye for 22 weeks, the whole grain rye significantly reduced body weight only at weeks 18 and 19 and lowered plasma leptin at the end of the trial, compared to whole grain wheat, despite no differences in energy intake [25]. However, several methodological differences limit the comparability to our study, including the use of a different species, a longer intervention duration, the absence of a control group, the use of a non‐obese model, differences in the intervention products composition (whole grain vs. refined wheat), and the food structure differences influenced by the use of cake‐based versus pelletized diets.

In randomized controlled human studies, rye products have been reported to promote subjective appetite and satiety for up to 8 h post‐consumption compared to refined or sifted wheat [26, 27, 28, 29]. However, these subjective effects were not supported by objective biomarkers of appetite and satiety, such as circulating leptin and ghrelin. Two studies reported no significant differences in overall postprandial ghrelin responses between whole grain rye and refined wheat interventions [30, 31]. In line with these findings, our rat study also showed reduced energy intake following rye consumption, compared to the control, high‐fat, and high‐fat→control groups, without corresponding changes in circulating leptin or ghrelin concentrations. It is plausible that the discrepancy between subjective and physiological outcomes, observed in both our study and the literature, may be attributed to the bulking and gastric distention effects of high‐fiber rye, which provide a sense of satiation. Such effects may influence subjective perceptions of fullness, reduce self‐reported appetite, and energy intake, but do not necessarily translate into measurable physiological changes in appetite‐regulating hormones.

All values for fasting blood glucose and fasting plasma insulin were within the normal healthy range and did not significantly differ between groups (Table 2). Similarly, a high‐fat diet composed of 60% energy from lard did not increase fasting glycemia in Sprague Dawley rats after 8 weeks of feeding [32]. A longer intervention using a 45% fat diet over 17 weeks likewise did not significantly affect fasting glucose concentrations in Sprague‐Dawley rats, although it resulted in delayed glucose clearance following an oral glucose tolerance test [22]. Interestingly, the same study reported a more pronounced decrease in glucose tolerance and insulin sensitivity in Wistar rats under comparable dietary conditions, suggesting strain‐specific differences in susceptibility to metabolic disturbances induced by a high‐fat diet. These findings confirm that Sprague‐Dawley rats may exhibit only moderate metabolic complications, including in glucose metabolism, following high‐fat feeding.

Although the glucose‐modulating effects of rye have been extensively studied in humans, limited research is available in rodent models. A study in Zucker diabetic fatty rats (ZDF‐*Lepr* ^fa^/Crl) found that whole grain rye bread lowered fasting blood glucose and HbA1c, and increased plasma insulin compared to refined wheat bread, indicating a delay in the onset of diabetes mellitus [33]. However, several factors limit direct comparability with our study. The ZDF rat model carries a mutation in the leptin receptor gene, resulting in a more severe and early‐onset form of obesity and metabolic complications, including hyperglycemia, compared to our model. Additionally, breads in that study were freeze‐dried, ground, mixed with 20% chow, and re‐pelletized, likely altering the food structure and its physiological effects. The bread also made up a higher proportion of the diet. In contrast, our rats were fed commercially available crispbreads provided as one‐third of total daily feed intake to preserve their natural structure and to better mimic habitual human dietary patterns, representing a key strength of our study design. Randomized‐controlled trials in humans have shown that rye may lower insulin concentrations without affecting systemic glucose, a phenomenon referred to as the “rye factor” [34]. In Wistar rats, feeding a high‐fat diet with or without fermented rye bread resulted in elevated fasting glucose in both groups, indicating that rye did not reduce the metabolic impact of the high‐fat diet [35]. Similarly, in C57BL/6N mice, fed a Western‐type diet for 17 weeks, rye bran‐feeding did not improve fasting glycemia and insulinemia, nor the postprandial glucose response to a glucose tolerance test, indicating that rye feeding does not improve glucose response in mice [36].

In humans, on the other hand, postprandial glucose response was reduced in healthy adults on a whole grain rye diet compared to a refined wheat diet [30, 37], suggesting that species differences between rodents and humans exist.

### 3.3 Plasma and Liver Lipids

**FIGURE 5:**
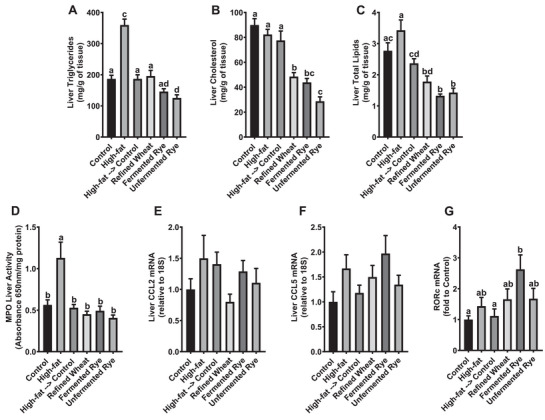
Hepatic lipids concentrations and inflammation at the end of the trial. Mean concentrations (mg/g tissue) of hepatic triacylglycerols (A), cholesterol (B), and total lipids (C); hepatic inflammatory markers, myeloperoxidase (MPO) activity (D), chemokine (C‐C motif) ligand 2 (CCL2) (E), chemokine (C‐C motif) ligand 5 (CCL5) (F), and retinoic acid‐related orphan receptor C (RORc) (G). Rats (*n* = 54) were acclimatized on a standard rat diet for 2 weeks and then randomly assigned to a control (*n* = 9) or high‐fat diet (*n* = 45) for 16 weeks. Then the animals in the high‐fat group were randomized to continue the high‐fat diet (*n* = 9) or to receive a standard diet alone (*n* = 9), or the standard diet with either refined wheat crispbread (*n* = 9), fermented rye crispbread (*n* = 9) or unfermented rye crispbread (*n* = 9) for 8 weeks. Data are presented as mean ± SEM from nine biological replicates (individual rats) per group. Statistical analysis was performed using repeated measures ANOVA with Tukey post‐hoc test. Bars not sharing a superscript letter are significantly different at *p* < 0.05.

Plasma concentrations of triacylglycerols, total cholesterol, and non‐HDL cholesterol did not differ between groups (Table 2). Plasma HDL cholesterol concentrations were significantly higher in rats consuming unfermented rye crispbread compared to the control, high‐fat, and high‐fat→control groups, but did not differ between the remaining groups (Table 2). The high‐fat diet group had significantly higher hepatic triacylglycerols compared to all other groups, and feeding unfermented rye crispbread further reduced hepatic triacylglycerols compared to the control, high‐fat, high‐fat→control, and refined wheat groups (Figure 5A). Liver cholesterol concentrations were similar in the control, high‐fat, and high‐fat→control groups and significantly lower in all three groups fed crispbreads (Figure 5B). Hepatic cholesterol was lowest in the rats fed unfermented rye crispbread and significantly lower than in the refined wheat group (Figure 5B). Total hepatic lipid concentrations were numerically, but not significantly, higher in the high‐fat compared to the control group (Figure 5C). Switching from the high‐fat to the control diet with additional crispbread or without (high‐fat→control) significantly reduced total liver lipids compared to the high‐fat diet, with the fermented and the unfermented rye crispbread groups presenting the lowest contents, which were significantly lower than those in all other groups except the refined wheat group (Figure 5C).

Similar to our observations, the feeding of rye crispbread, compared to a fiber‐free control, significantly reduced plasma and hepatic cholesterol in Wistar rats fed a diet with cholesterol and cholic acid to induce hypercholesterolemia, which the authors attributed to reduced enterohepatic cholesterol and bile acid reabsorption [38]. Unfermented rye crispbread exhibited the most pronounced effects on lipid metabolism in our rats, potentially due to its more structurally intact fiber matrix and the larger fragments of aleurone layers compared to the refined wheat and fermented rye [6]. However, it is important to note that the differences between fermented and unfermented rye groups were not statistically significant. Despite the species differences, our findings are in agreement with data from a randomized controlled trial, which found that rye bread consumption reduced plasma LDL‐cholesterol without affecting plasma HDL‐cholesterol or triacylglycerol concentrations [8].

### 3.4 Hepatic Inflammation

Myeloperoxidase (MPO) is a proinflammatory enzyme released by neutrophils [39]. Hepatic MPO activity significantly increased in animals fed a high‐fat diet compared to control rats, and switching from the high‐fat to the standard diet alone (high‐fat→control) or in combination with crispbreads significantly reduced MPO activity back to the level observed in control animals (Figure 5D). This indicates that neutrophil‐driven inflammation was induced by high‐fat feeding and attenuated following the dietary shifts. CCL2 and CCL5 are chemokines that recruit immune cells to sites of tissue inflammation or injury [40]. The high‐fat diet did not significantly change the relative mRNA of chemoattractant chemokines CCL2 (Figure 5E) and CCL5 (Figure 5F) compared to control, suggesting that hepatic immune cell infiltration was not markedly induced. An increased relative mRNA expression of RORc was observed in rats fed fermented rye crispbread compared to the control and high‐fat groups (Figure 5G). This effect may be incidental since no significant changes were observed in other inflammatory markers of the liver. Our findings indicate that switching from a high‐fat to a lower‐calorie diet was sufficient to reduce hepatic inflammation in rats. Consequently, supplementation with fermented or unfermented rye crispbreads or refined wheat crispreads did not confer additional benefits. These results are consistent with previous findings in Sprague‐Dawley rats, which showed that switching from a 23‐week high‐fat diet to a 20‐week standard diet prevented the progression of nonalcoholic steatohepatitis, compared to continuous high‐fat feeding for 43 weeks, although hepatic steatosis persisted [41].

### 3.5 Biomarkers of Inflammation, Gut Integrity, and Permeability in the Colon

**FIGURE 6:**
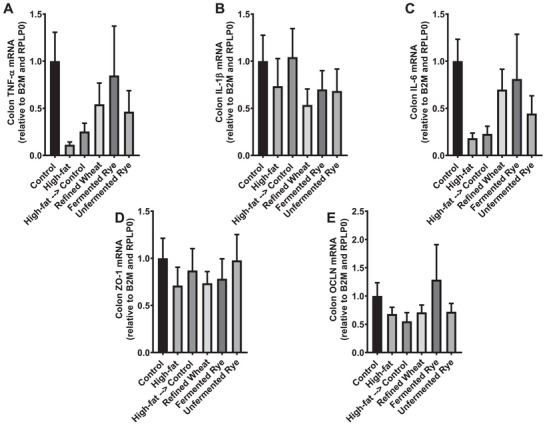
Markers of colon inflammation and integrity at the end of the trial. Mean mRNA expression of cytokines, tumor necrosis factor alpha (TNF‐α) (A), interleukin 1 beta (IL‐1β) (B), interleukin‐6 (IL‐6) (C); and tight junction proteins, zonula occludens (ZO‐1) (D) and occludin (OCLN) (E), normalized to ribosomal protein lateral stalk subunit P0 (RPLP0) and Beta‐2‐Microglobulin (B2M). Rats (*n* = 54) were acclimatized on a standard rat diet for 2 weeks and then randomly assigned to a control (*n* = 9) or high‐fat diet (*n* = 45) for 16 weeks. Then the animals in the high‐fat group were randomized to continue the high‐fat diet (*n* = 9) or to receive a standard diet alone (*n* = 9), or the standard diet with either refined wheat crispbread (*n* = 9), fermented rye crispbread (*n* = 9) or unfermented rye crispbread (*n* = 9) for 8 weeks. Data are presented as mean ± SEM from nine biological replicates (individual rats) per group. Each PCR sample was measured in triplicate (technical replicates). Statistical analysis was performed using one‐way ANOVA with Tukey post‐hoc test and significance accepted at *p* < 0.05.

No significant differences between groups, but a high variability in relative mRNA expression of TNF‐α, IL‐1β, and IL‐6, and of ZO‐1 and OCLN (Figure 6) was observed in the colon. Plasma concentrations of the permeability marker lipopolysaccharide (LPS) did not differ between groups and remained within the normal healthy range for rats (Table 2). This study is the first to investigate local inflammation and gut barrier integrity in response to fermented and unfermented rye crispbread compared to refined wheat locally in the colon. Prior research has focused on the effects of rye on systemic inflammation [4, 30, 34]. However, diet may also exert local effects on the gut that warrant further investigation.

A deeper understanding of the metabolic advantages of whole grain rye, compared to refined wheat in staple foods, such as bread, and how processing techniques like fermentation modulate these effects, is essential for building robust evidence for dietary recommendations. Nutrition research on rye has primarily focused on its impact on satiety and glycemic responses [8, 9, 30, 31, 37, 42, 43, 44, 45, 46], however, our study shows that whole grain rye bread, particularly the unfermented, further exhibits lipid‐lowering effects. Since crispbread intake did not differ between groups, the observed metabolic outcomes are likely due to the crispbread itself, rather than to differences in total energy intake or other confounding factors. These findings may be attributed to the rye distinctive fiber content, their physicochemical properties, and their digestion kinetics compared to wheat. Particularly the viscous and soluble arabinoxylans in rye that may delay gastric emptying and have the capacity to encapsulate nutrients to reduce the diffusion rate of enzymes and nutrients in the digesta, thereby decreasing digestion and absorption of simple sugars and lipids, as well as the reabsorption of bile acids [47]. Further, the (poly)phenol‐rich bioactive compounds are higher in rye compared to wheat, and retained in unfermented bread, but some may be degraded or structurally modified during fermentation [4, 6, 48, 49]. However, confirming these mechanisms requires further research in models that are more responsive to diet‐inducing obesity. Although Sprague‐Dawley rats respond similarly to humans in many metabolic outcomes and feeding cues, differences in the digestion and fermentation of fibers may influence the production of microbial metabolites and their systemic effects. Therefore, targeted human intervention studies are warranted to better characterize the lipid‐modulating potential of whole grain rye and clarify the role of fermentation in shaping these responses.

### 3.6 Concluding Remarks

In the present experiment, the high‐fat diet increased adiposity and plasma leptin concentration in rats, without significant changes in body weight, which contributed to metabolic disturbances, such as elevated hepatic lipids and mild neutrophil‐driven inflammation. Some effects, such as hepatic cholesterol, persisted after switching to a lower‐caloric diet. However, adding unfermented rye crispbread, amounting to one third of the daily feed intake, to the lower‐energy diet increased plasma HDL cholesterol and reduced hepatic triacylglycerol and cholesterol concentrations, compared to refined wheat crispbread, thus highlighting potentially important metabolic benefits. No significant differences in metabolic outcomes were observed between fermented and unfermented rye. Further studies using animal models with stronger diet‐induced obesity responses are warranted to investigate the underlying mechanisms behind observed effects in human studies of unfermented high fiber rye on body weight regulation, biomarkers of appetite, and glucose homeostasis. Human intervention studies are also warranted to confirm our findings on the potential lipid‐modulating effect of whole grain rye and elucidate the impact of fermentation in shaping these responses.

## Conflicts of Interest

R.L. is the founder of the Nordic Rye Forum, which is a research and dissemination platform for research related to rye and health that includes academic institutions as well as institutes and the food industry with an interest in rye across the Nordic region. The forum and its activities are funded by the industrial partners. R.L. is the PI of several projects funded by several cereal industrial companies. Such funding is used to carry out scientific studies. The remaining authors declare no conflicts of interest.

## Supporting information




**Supporting File**: mnfr70352‐sup‐0001‐SuppMat.docx.

## Data Availability

The data that support the findings of this study are available from the corresponding author upon reasonable request.
