# Supplementary material for: Unfermented High‐Fiber Rye Crispbread Increases Plasma HDL and Reduces Hepatic Lipids Compared to Refined Wheat in Rats Fed a High‐Fat Diet
Source: Mol Nutr Food Res. 2025 Dec 13;70(1):e70352. doi: 10.1002/mnfr.70352 (PMC12728384; doi:10.1002/mnfr.70352)
Supplement: Supplementary file 1 — Supporting File: mnfr70352‐sup‐0001‐SuppMat.docx. [file MNFR-70-e70352-s001.docx]

**Reverse transcription quantitative real-time polymerase chain reaction (RT-qPCR) for liver tissues**

Total RNA was isolated from liver tissue using the Animal Tissue RNA Purification Kit (#25700, Norgen Biotek Corporation, Thorold, ON, Canada). Approximately 10 mg of liver tissue were homogenized in 600 µL of RL buffer (supplied with the kit) and β-mercaptoethanol using the gentle-MACS^TM^ tissue dissociator. The homogenate was centrifuged and 600 µL of 70% ethanol were added to the supernatant. RNA was then bound to the purification column, followed by three successive washes and eluted in accordance with the manufacturer’s protocol. The purified RNA samples were stored at -80 °C. RNA concentration was determined using a NanoDrop™ One/OneC Microvolume UV-Vis Spectrophotometer (Thermo Fisher, Waltham, MA, USA). A 1 µL aliquot of the sample was applied to the instrument and absorbance read at 280 nm. To assess the purity of the extracted RNA, the ratio of absorbance at 260 nm to 280 nm was calculated, with an acceptable range between 1.9 and 2.1. Subsequently, 1000 ng of RNA were reverse-transcribed into complementary DNA (cDNA) using the SensiFAST™ cDNA Synthesis Kit 50 reactions (#BIO-65054 Meridian Bioscience, Cincinnati, Ohio, USA). The mix was prepared on ice, mixing 5xtransAmp buffer and reverse transcriptase in a 4:1 ratio. In 200 µL-tube, RNase-free water was added, followed by samples and the above-mentioned mix to reach a final volume of 20 μL, as reported in **Supplementary Table 1**:

**Supplementary Table 1. Composition of the mix for the retro transcription of mRNA into cDNA**

| **Reagent** | **Volume (μl)** |
| --- | --- |
| Total RNA/mRNA | *N* μl (1000 ng) |
| 5x transamp buffer | 4 μl |
| Reverse transcriptase | 1 μl |
| DNase/RNase-free water | Up to 20 μl |

The following program was set up in a thermal cycler (CFX Connect Real-Time PCR Detection System, Bio-Rad, Hercules, CA, USA):

- 25 °C for 10 min (primer annealing)
- 42 °C for 15 min (reverse transcription)
- 85 °C for 5 min (inactivation)

Once the retro transcription was concluded, cDNA was stored at -20° C.

Real time qPCR was performed using 50 ng of cDNA (liver) and the SensiFAST™ SYBR® No-ROX Kit (#BIO-98020 Meridian Bioscience, Cincinnati, Ohio, USA). A mix with the provided master mix, primers and RNAase free water was prepared. QuantiTect primers for 18s (#QT02589300), CCL2 (#QT00183253) and CCL5 (#QT01083614) used in this study were purchased from Qiagen (Hilden, Germany). qRT-PCR reaction was carried out on the same thermocycler utilized for the retro transcription phase. Relative gene expression was obtained after normalization to the housekeeping gene (18s) using the formula 2^-ΔΔCT^.

Reactions were performed using the conditions reported in **Supplementary Table 2**:

**Supplementary Table 2. Experimental conditions used for the qRT-PCR**

| **Cycles** | **Temperature (°C)** | **Time** | **Phase** |
| --- | --- | --- | --- |
| 1 | 95 | 2 min | Polymerase Activation |
| 40 | 95  60  72 | 5 sec  10 sec  13 sec | Denaturation  Annealing  Extension |

**RT-qPCR for colon tissues**

Colonic expression of tumor necrosis factor alpha (TNF-α), interleukin-1β (IL-1β), interleukin-6 (IL-6), zonulin-1 (ZO-1), and occludin (OCLN) was analyzed by reverse transcription quantitative real-time polymerase chain reaction (RT-qPCR). In brief, RNA was extracted from the colon tissues (20-25 mg) according to the manufacturer‘s instructions (RNeasy^®^ Plus Mini Kit, Cat. 74136; QIAGEN, Germany). The RNA concentration and purity were measured with Agilent BiotTek Take 3 (Agilent Technologies, Germany). For normalization, RNA was diluted to an amount of 1 µg of RNA, then synthesized to cDNA (Cat. 1708891; Bio-Rad Laboratories, Germany). The PCR reaction was performed using the CFX 96 Real-Time PCR Detection Systems (Bio-Rad Laboratories, Germany) and set at the following respective steps: 95 °C (10 s; denaturation), 55-68 °C (15 s; primer annealing), and 72 °C (29 s; elongation) for a total of 40 cycles. The total reaction volume was 10 µL (iQ SYBR^®^ Green Supermix, Cat. 1708882; Bio-Rad, Germany). The annealing temperature of the primers (**Supplementary Table 3**) was validated and optimized based on the PCR product size and agarose gel electrophoresis results. The gel DNA was extracted following the instructions of the manufacturer (QIAquick Gel Extraction Kit, Cat. 28704; Qiagen, Germany) and subsequently used to prepare a serial dilution for the standard curve. All samples were analyzed in triplicate and relative mRNA expression was normalized using the geometric mean of the reference genes RPLP0 and B2M. All of the RT-qPCR protocol was carried in accordance with the Minimum Information for Publication of Quantitative Real-Time PCR Experiments (MIQE) guidelines (Bustin et al., 2009).

**Supplementary Table 3. Primer sequences used in the RT-qPCR analysis**

| Gene | Primer sequence (5’- 3’) | Annealing Temperature (°C) | PCR product size (bp) | Length of bases | Reference | Gene Code |  |
| --- | --- | --- | --- | --- | --- | --- | --- |
| TNF-α | F: GCCTCCTCTCTGCCATCAAG  R: CTCCAAAGTAGACCTGCCCG | 66 | 186 | 20 | (Guo et al., 2022) | NM_012675.3 |  |
| IL-1β | F: CCTTGTGCAAGTGTCTGAAG  R: GGGCTTGGAAGCAATCCTTA | 60 | 137 | 20 | (Frank et al., 2016) | NM_031512.2 |  |
| IL-6 | F: CCGGAGAGGAGACTTCACAGAGGA  R: AGCCTCCGACTTGTGAAGTGGTATA | 66 | 71 | 24 | (de Melo et al., 2015) | NM_012589.2 |  |
| ZO-1 | F: CAGGCCATTACGAGCCTCTC  R: GGCTGTGGCTTGGTAGCTG | 68 | 104 | 20 | (Li et al., 2020) | NM_001106266.1 |  |
| OCLN | F: CATCGCTTCCTTGGTGATCT  R: CAGGATTGCGCTGACTATGA | 66 | 100 | 20 | (Lee et al., 2015) | AB016425.1 |  |
| RPLP0 | F: CCCTTCTCCTTCGGGCTGAT  R: TGAGGCAACAGTCGGGTAGC | 66 | 165 | 20 | (Lu et al., 2021) | NM_022402.2 |  |
| B2M | F: CGAGACCGATGTATATGCTTGC  R: GTCCAGATGATTCAGAGCTCCA | 60 | 114 | 22 | (Toval et al., 2020) | NM_012512.2 |  |
| F: forward primer; R: reverse primer; TNF-α: tumor necrosis factor alpha; IL-1β: interleukin-1β; IL-6: interleukin-6; ZO-1: zonula occludens; OCLN: occludin; RPLP0: ribosomal protein lateral stalk subunit P0; B2M: beta-2-microglobulin; PCR: polymerase chain reaction; bp: number of base pairs. | | | | | | | |

**Supplementary Figure 1.** Daily feed and bread intake (gram per cage). Rats (n=54) were acclimatized on a standard rat diet for 2 weeks and then randomly assigned to a control (n=9) or high-fat diet (n=45) for 16 weeks. Then the animals in the high-fat group were randomized to continue the high-fat diet (n=9) or to receive a standard diet alone (n=9), or the standard diet with either refined wheat crispbread (n=9), fermented rye crispbread (n=9) or unfermented rye crispbread (n=9) for 8 weeks. Data are presented as mean ± SEM. Bars not sharing a superscript letter are significantly different at P>0.05.

1. Bustin, S. A., Benes, V., Garson, J. A., Hellemans, J., Huggett, J., Kubista, M., Mueller, R., Nolan, T., Pfaffl, M. W., Shipley, G. L., Vandesompele, J., & Wittwer, C. T. (2009). The MIQE Guidelines: Minimum Information for Publication of Quantitative Real-Time PCR Experiments. *Clinical Chemistry*, *55*(4), 611–622. https://doi.org/10.1373/clinchem.2008.112797
2. de Melo, J. O., Soto, S. F., Katayama, I. A., Wenceslau, C. F., Pires, A. G., Veras, M. M., Furukawa, L. N. S., de Castro, I., Saldiva, P. H. N., & Heimann, J. C. (2015). Inhalation of fine particulate matter during pregnancy increased IL-4 cytokine levels in the fetal portion of the placenta. *Toxicology Letters*, *232*(2), 475–480. https://doi.org/10.1016/j.toxlet.2014.12.001
3. Frank, M. G., Weber, M. D., Fonken, L. K., Hershman, S. A., Watkins, L. R., & Maier, S. F. (2016). The redox state of the alarmin HMGB1 is a pivotal factor in neuroinflammatory and microglial priming: A role for the NLRP3 inflammasome. *Brain, Behavior, and Immunity*, *55*, 215–224. https://doi.org/10.1016/j.bbi.2015.10.009
4. Guo, G., Kong, Y., Su, J., Wang, G., Zhang, M., Wang, S., & Song, Z. (2022). Immunomodulatory activity of aqueous extract from Crassostrea sikamea in the splenocytes of Sprague‐Dawley rats. *Food Science & Nutrition*, *10*(3), 813–821. https://doi.org/10.1002/fsn3.2710
5. Lee, S.-M., Han, H. W., & Yim, S. Y. (2015). Beneficial effects of soy milk and fiber on high cholesterol diet-induced alteration of gut microbiota and inflammatory gene expression in rats. *Food & Function*, *6*(2), 492–500. https://doi.org/10.1039/C4FO00731J
6. Li, Y., Guo, R., Zhang, M., Chen, P., Li, J., & Sun, Y. (2020). Protective effect of emodin on intestinal epithelial tight junction barrier integrity in rats with sepsis induced by cecal ligation and puncture. *Experimental and Therapeutic Medicine*, *19*(6), 3521–3530. https://doi.org/10.3892/etm.2020.8625
7. Lu, X., Liu, Y., Zhang, D., Liu, K., Wang, Q., & Wang, H. (2021). Determination of the panel of reference genes for quantitative real-time PCR in fetal and adult rat intestines. *Reproductive Toxicology*, *104*, 68–75. https://doi.org/10.1016/j.reprotox.2021.07.001
8. Toval, A., Vicente-Conesa, F., Martínez-Ortega, P., Kutsenko, Y., Morales-Delgado, N., Garrigos, D., Alonso, A., Ribeiro Do Couto, B., Popović, M., & Ferran, J. L. (2020). Hypothalamic Crh/Avp, Plasmatic Glucose and Lactate Remain Unchanged During Habituation to Forced Exercise. *Frontiers in Physiology*, *11*, 410. https://doi.org/10.3389/fphys.2020.00410
